# Identification of Hub mRNAs and lncRNAs in Atrial Fibrillation Using Weighted Co-expression Network Analysis With RNA-Seq Data

**DOI:** 10.3389/fcell.2021.722671

**Published:** 2021-10-04

**Authors:** Pan Yang, Yujing Cao, Huagang Jian, Hao Chen

**Affiliations:** ^1^Emergency Department, The Second Affiliated Hospital of Chongqing Medical University, Chongqing, China; ^2^Department of Cardiovascular Surgery, Chongqing General Hospital, University of Chinese Academy of Sciences, Chongqing, China; ^3^Department of Cardiology, The Second Affiliated Hospital of Chongqing Medical University, Chongqing, China

**Keywords:** atrial fibrillation, WGCNA, bioinformatics analysis, PPI network, RNA sequencing

## Abstract

Atrial fibrillation (AF)/paroxysmal AF (PAF) is the main cause of cardiogenic embolism. In recent years, the progression from paroxysmal AF to persistent AF has attracted more and more attention. However, the molecular mechanism of the progression of AF is unclear. In this study, we performed RNA sequencing for normal samples, paroxysmal AF and persistent AF samples to identify differentially expressed gene (DEG) and explore the roles of these DEGs in AF. Totally, 272 differently expressed mRNAs (DEmRNAs) and 286 differentially expressed lncRNAs (DElncRNAs) were identified in paroxysmal AF compared to normal samples; 324 DEmRNAs and 258 DElncRNAs were found in persistent atrial fibrillation compared with normal samples; and 520 DEmRNAs and 414 DElncRNAs were identified in persistent AF compared to paroxysmal AF samples. Interestingly, among the DEGs, approximately 50% were coding genes and around 50% were non-coding RNAs, suggesting that lncRNAs may also have a crucial role in the progression of AF. Bioinformatics analysis demonstrated that these DEGs were significantly related to regulating multiple AF associated pathways, such as the regulation of vascular endothelial growth factor production and binding to the CXCR chemokine receptor. Furthermore, weighted gene co-expression network analysis (WGCNA) was conducted to identify key modules and hub RNAs and lncRNAs to determine their potential associations with AF. Five hub modules were identified in the progression of AF, including blue, brown, gray, turquoise and yellow modules. Interestingly, blue module and turquoise module were significantly negatively and positively correlated to the progression of AF respectively, indicating that they may have a more important role in the AF. Moreover, the hub protein-protein interaction (PPI) networks and lncRNA–mRNA regulatory network were constructed. Bioinformatics analysis on the hub PPI network in turquoise was involved in regulating immune response related signaling, such as leukocyte chemotaxis, macrophage activation, and positive regulation of α-β T cell activation. Our findings could clarify the underlying molecular changes associated fibrillation, and provide a useful resource for identifying AF marker.

## Introduction

Atrial fibrillation (AF) is a common tachyarrhythmia, which had been the main cause of cardiogenic embolic infarction (Abdul-Rahim et al., 2015; Kelley, 2015). AF develop from paroxysmal AF to persistent AF (Shukla and Curtis, 2014). Paroxysmal AF occurs in about 50% of all AF cases (Skaarup et al., 2016). Persistent AF occurs in about 20% of chronic heart failure and is related to a poor prognosis (Cornelis et al., 2018). In the past 10 years, AF ablation has been a common treatment of AF (Pelargonio et al., 2021). Previous studies have shown that AF is a complex disease caused by genetic and environmental factors (Lubitz et al., 2010). In the past few decades, some regulators related to AF have been discovered, such as NLRP3 (Yao et al., 2018), JPH2 (Beavers et al., 2013) and microRNA-26 (miR-26) (Luo et al., 2013). Knockout of NLRP3 inhibits the development of AF (Yao et al., 2018). MicroRNA-26 regulates AF and promotes changes in the inward rectifier potassium current of AF (Luo et al., 2013). However, the underlying mechanism of the progression of AF remains unclear. Understanding the molecular mechanism of AF will help to identify biomarkers for the early diagnosis and treatment for AF.

LncRNA is a set of non-coding transcripts longer than 200 nucleotides (Shi et al., 2020a; Statello et al., 2021). In recent years, lncRNA has been confirmed to have a crucial role in a variety of cell functions, including epigenetic regulation, transcription regulation, etc., and has been potential biomarkers for disease diagnosis and treatment (Cao, 2014; Gu and Chen, 2020). In human cells, it has been identified more than 100,000 lncRNAs (Statello et al., 2021), which play an important role in the cardiovascular system. However, only a small number of lncRNA functions have been studied in AF. For example, in AF, the lncRNA LICPAR modulates atrial fibrosis through Smad signaling (Wang et al., 2020). LncRNA NEAT1 regulates atrial fibrosis during AF through the miR-320/Npas2 axis (Dai et al., 2021). LncRNA-MIAT regulates AF-induced myocardial fibrosis through miR-133a-3p (Yao et al., 2020). LncRNA-PVT1 modulates atrial fibrosis during AF through miR-128 (Cao et al., 2019). To understand the expression patterns and possible functions of lncRNAs in AF could provide helpful information for the treatment of AF.

Weighted gene co-expression network analysis (WGCNA) is used to cluster highly related genes to further understand the hub modules and disease types/clinical phenotypes (Langfelder and Horvath, 2008). In recent years, WGCNA has been used to identify key regulators in disease progression. For example, Ren et al. (2021) performed WGCNA analysis to identify diagnostic genes and important microRNAs associated with rheumatoid arthritis. Here, we performed RNA sequencing to identify differently expressed mRNAs (DEmRNAs) and lncRNAs in normal samples, paroxysmal AF, and persistent AF. In addition, we use bioinformatics methods, such as WGCNA, and PPI network analysis, to identify the hub lncRNAs and mRNAs in AF. Our findings aim to clarify the underlying molecular changes associated fibrillation, and provide a useful resource for identifying AF marker.

## Materials and Methods

### RNA-Seq Analysis

In this study, 10 control samples, 10 paroxysmal AF, and 10 persistent AF samples were prepared for RNA sequencing. RNA was extracted from Approximately 500 mg AF and normal samples using the RNeasy mini kit (QIAGEN).

We next applied Corall Total RNA Seq library preparation kit (Lexogen, Vienna, Austria) for RNA Seq library using 150 ng of total RNA. The RiboCop rRNA Depletion Kit (Lexogen, Vienna, Austria) was used to remove rRNA. The quality of the sequencing library was evaluated by D1000 screen tape analysis using the 4200 TapeStation system (Agilent, United States) and quantified using QubitTM dsDNA HS analysis kit (Invitrogen, United States). RNA processing was used by Illumina NextSeq 500 sequencing. The R software package Deseq2 was used for mRNA differential expression analysis (Zhang et al., 2020). The genes with |log2 fold change | ≥ 1 and FDR ≤ 0.1 were considered to be differentially expressed (Gu et al., 2021a,b).

### Weighted Gene Co-expression Network Construction

A scale-free co-expression network was constructed by using WGCNA package in R (Langfelder and Horvath, 2008). The appropriate soft threshold power (β) is determined based on a scale-free topology criterion. The result was clustered by topological overlap matrix analysis. In addition, the correlations between module eigengenes (MEs) were calculated via Pearson’s correlation analysis.

### Functional Annotations

In order to explore the functional annotation of DEGs, we performed gene ontology (GO) (Gu et al., 2020a), kyoto encyclopedia of genes and genomes (KEGG) (Gu et al., 2020b; Liang et al., 2020) and UniProt analysis to predict gene functions using DAVID system (Jiao et al., 2012; Shi et al., 2020b).

### PPI Network Construction

We used the STRING to construct a PPI network (Shi et al., 2018). The PPI network was visualized through Cytoscape, and further filtered through plug-in molecular complex detection (MCODE) to determine the candidate hub differentially expressed module (DEM) (Feng et al., 2019). The biological significance of gene modules was visualized with MCODE in Cytoscape to identify the most significant module (Gu et al., 2020c).

### The Co-expression Network Analysis of Hub lncRNAs

Cytoscape displays the co-expression network of hub lncRNAs through the topological analysis of lncRNAs, the central nodes of these networks were explored. The significantly co-expression network of hub lncRNA-mRNAs with an absolute Pearson correlation coefficient >0.99 was chosen as the targets to build the network.

The WGCNA package in R was used to generate a co-expression network of DEGs (Langfelder and Horvath, 2008). Pearson’s correlation analysis was conducted as a similarity measure. The soft threshold power (β) of the correlation matrix was used to emphasize strong correlations between genes and penalize weak correlations. Next, the adjacency was used to calculate the topological overlap matrix (TOM).

## Results

### Identification of DEGs Among Normal, Paroxysmal AF, and Persistent AF Samples

**FIGURE 1:**
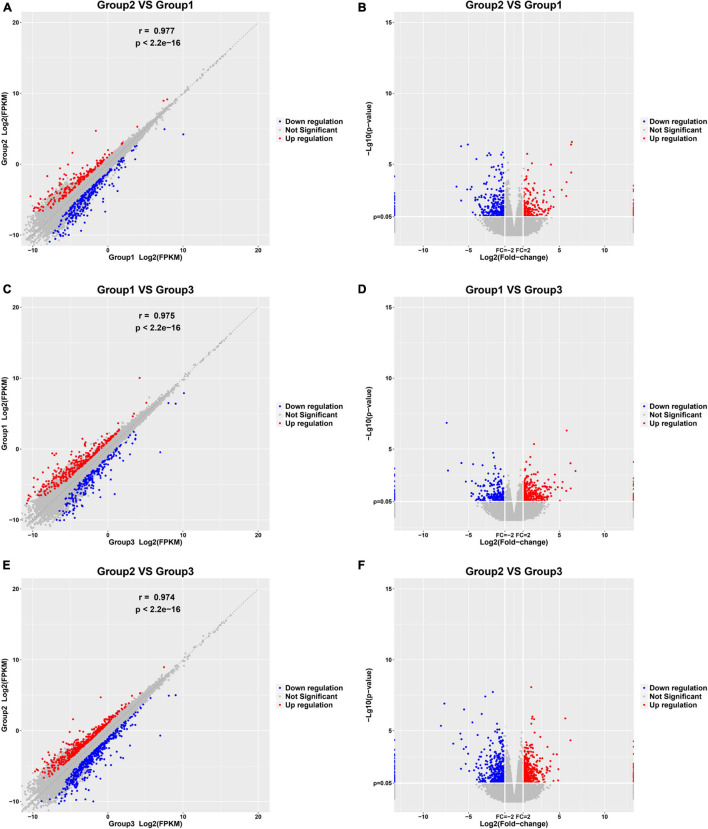
Identification of DEGs among normal, paroxysmal AF and persistent AF samples. **(A,B)** The scatter plot and volcano plot show the DEGs in paroxysmal AF compared to normal samples. **(C,D)** The scatter plot and volcano plot show the DEGs in persistent AF compared to normal samples. **(E,F)** The scatter plot and volcano plot show the DEGs in persistent AF compared to paroxysmal AF samples.

In this study, 10 control samples, 10 paroxysmal AF, and 10 persistent AF samples were sequenced among their RNA. Then, we identified DEGs in the progression of AF with the R package Limma. 558 genes were identified to be differently expressed in paroxysmal AF compared to control samples (Figures 1A,B); 582 genes were identified to be differently expressed in persistent AF compared to control samples (Figures 1C,D); and 934 genes were identified to be differently expressed in persistent AF compared to paroxysmal AF tissues (Figures 1E,F). The heat map and volcano map of the DEG are shown in Figure 1.

**FIGURE 2:**
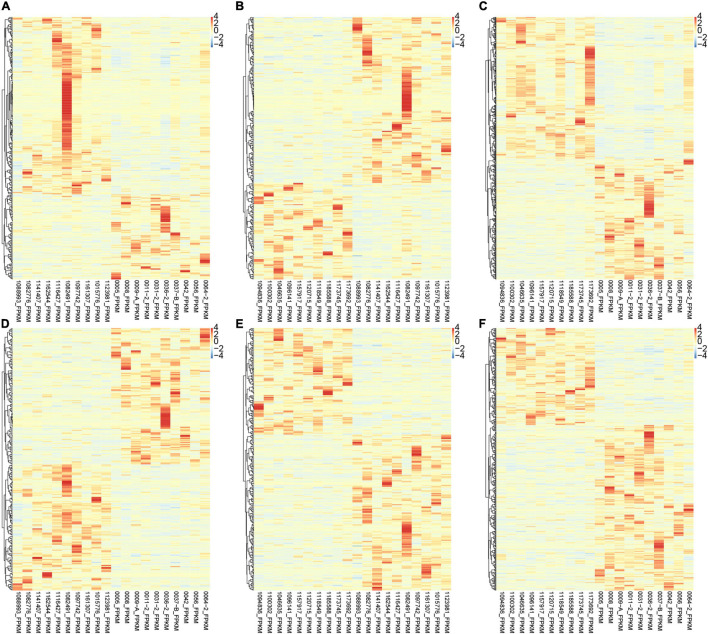
Identification of DEmRNAs and lncRNAs among normal, paroxysmal AF and persistent AF samples. **(A–C)** The heat map shows the DEmRNAs in paroxysmal AF compared to normal samples **(A)**, persistent AF compared to normal samples **(B)**, and persistent AF compared to paroxysmal AF samples **(C)**. **(D–F)** The heat map shows the differently expressed lncRNAs in paroxysmal AF compared to normal samples **(A)**, persistent AF compared to normal samples **(B)**, and persistent AF compared to paroxysmal AF samples **(C)**.

Interestingly, we found that the expression of various lncRNAs in the progression of atrial fibrillation was differently changed. Among the DEGs, about 50% were coding genes and around 50% were non-coding RNAs (Figure 2). Compared with normal samples, 272 DEGs were found in patients with paroxysmal atrial fibrillation, including 100 upregulated and 172 downregulated mRNAs (Figure 2A). Overall, 324 DEmRNAs were found in persistent atrial fibrillation compared with normal samples, including 219 upregulated and 105 downregulated mRNAs (Figure 2B). In addition, compared with paroxysmal atrial fibrillation samples, 520 genes were identified in persistent atrial fibrillation samples, including 281 upregulated genes and 239 downregulated genes (Figure 2C).

Therefore, we also focused on the expression changes and potential functions in AF. As shown in Figure 1, compared with normal samples, a total of 286 DElncRNAs were identified in paroxysmal atrial fibrillation, and 116 lncRNAs were upregulated and 170 lncRNAs were downregulated (Figure 2D). Overall, compared with normal samples, 124 upregulated and 134 downregulated lncRNAs were identified in persistent atrial fibrillation (Figure 2E). In addition, compared with paroxysmal atrial fibrillation samples, 414 DElncRNAs were identified in persistent atrial fibrillation samples, including 153 upregulated lncRNAs and 261 downregulated lncRNAs (Figure 2F). Of note, several lncRNAs were observed to be differently expressed in multiple stages of AF, such as MTND1P23, RP11-1081M5.2, and XIST.

### GO Analysis of DEmRNAs Highlights Specific Processes-Involvement

**FIGURE 3:**
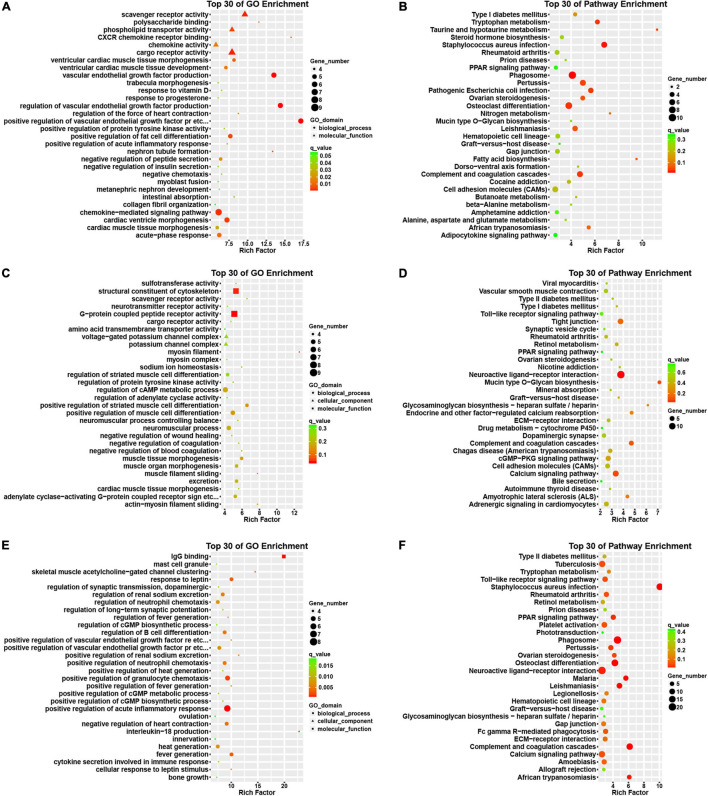
Bioinformatics analysis of DEGs among normal, paroxysmal AF and persistent AF samples. **(A,B)** GO and KEGG pathway analysis of DEmRNAs in paroxysmal AF compared to normal samples. **(C,D)** GO and KEGG pathway analysis of DEmRNAs in persistent AF compared to normal samples. **(E,F)** GO and KEGG pathway analysis of DEmRNAs in persistent AF compared to paroxysmal AF samples.

Then, we conducted an *in silico* analysis of the DEGs in the progression of AF. Interestingly, we found that the differentially expressed genes in patients with paroxysmal AF compared with control samples are mainly related to the positive regulation of VEGF production, CXCR chemokine receptor binding, VEGF production, and the formation of renal tubules (Figure 3A).

In addition, we also found that the genes differentially expressed between persistent AF and normal samples were mainly involved in the regulation of myosin filaments, myofilament sliding, actin-myosin filament sliding, scavenger receptor activity, sodium ion homeostasis, negatively regulating blood coagulation, muscle tissue morphogenesis, negatively regulating coagulation, myocardial tissue morphogenesis (Figure 3C).

Finally, we found that the differentially expressed genes between persistent AF and paroxysmal AF were mainly related to the production of interleukin-18 (IL-18), IgG binding, clustering of skeletal muscle acetylcholine-gated channels, positive regulation of renal sodium excretion, fever production, positive regulation of fever production, positive regulation of VEGFR signaling pathway, cell response to leptin stimulation, response to leptin, the regulation of fever, and the positive regulation of the chemotaxis of granulocytes (Figure 3E).

### Pathway Analysis of DEmRNAs Highlights Specific Processes-Involvement

Use the KEGG database to analyze the pathway of DEmRNAs. We found that the genes differentially expressed in paroxysmal AF and normal samples mainly involve phagosomes, whooping cough, pathogenic *Escherichia coli* infection, complement and coagulation cascade, osteoclast differentiation, tryptophan metabolism, taurine and subcutaneous Taurine metabolism, fatty acid biosynthesis, leishmaniasis, ovarian steroid production and cytokine-cytokine receptor interaction (Figure 3B).

The differentially expressed genes in persistent atrial fibrillation compared with normal atrial fibrillation mainly involved neuroactive ligand-receptor interactions, tight junctions, complement and coagulation cascades, mucin-type O-glycan biosynthesis, calcium signaling pathways, endocrine, and other factors regulating calcium reabsorption, glycosaminoglycan biosynthesis-heparan/heparin sulfate, amyotrophic lateral sclerosis (ALS), cGMP-PKG signaling pathway, and retinol metabolism (Figure 3D).

Compared with paroxysmal AF, the differentially expressed genes in persistent AF mainly involved phagosomes, complement and coagulation cascade, osteoclast differentiation, leishmaniasis, malaria, African trypanosomiasis, neuroactive ligand-receptor interaction, PPAR signaling pathway, whooping cough, and ovarian steroid hormone production (Figure 3F).

### Weighted Gene Co-expression Network Construction

**FIGURE 4:**
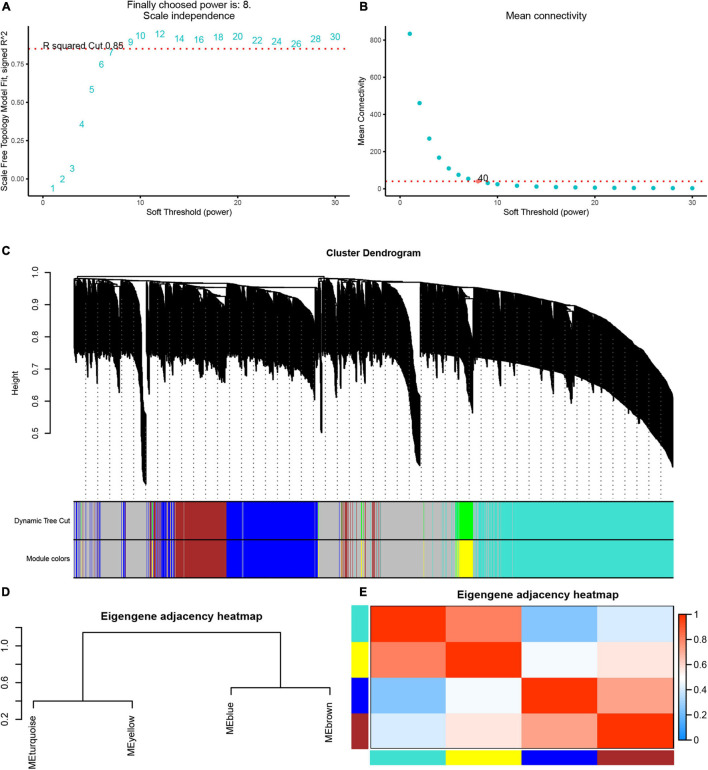
Weighted gene co-expression network analysis of differently expressed genes. **(A)** The scale-free fit index (left). **(B)** Mean connectivity (right) for various soft-thresholding powers. **(C)** Dendrogram of the DEGs clustered based on a dissimilarity measure (1-TOM). **(D)** Cluster analysis and heatmap of the genes in different modules. **(E)** Heatmap showing the relationship between module eigengenes.

Next, we used the WGCNA package in the R software to perform a co-expression network analysis of the gene expression in the progress of AF. In order to identify all co-expressed genes, we chose β = 8 (fit value *R*^2^ = 0.85) as the cutoff to build a network (Figures 4A,B).

Based on these analyses, we initially obtained five gene modules and then used the dynamic tree cutting algorithm in the WGCNA software package to process the hierarchical clustering tree. In the progress of AF, a total of four gene modules were obtained, including blue, brown, turquoise and yellow modules (Figure 4C). In addition, the gray module includes all genes that cannot be put into any other modules. The clustering tree diagram of the module is shown in the Figure 4D, while the intrinsic clustering of the modules is provided in Figure 4E.

**FIGURE 5:**
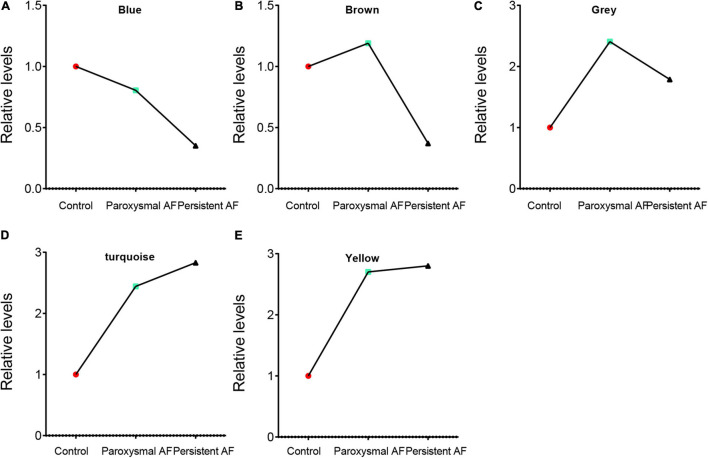
The analysis of the correlation between AF progression and module expression. **(A–E)** The analysis of the correlation between AF progression and the average expression of genes in blue module **(A)**, brown module **(B)**, grey module **(C)**, turquoise module **(D)**, and yellow module **(E)**.

We performed the first principal component analysis (PCA) on five gene modules. The PCA results reflected the main trend of gene expression in each module. Our results indicated that blue module was negatively correlated to the AF progression and downregulated in paroxysmal and persistent AF compared to control samples, and downregulated in persistent AF samples compared to paroxysmal AF samples (Figure 5A). The brown module was downregulated in persistent AF samples compared to control and paroxysmal AF samples, and not differently expressed in paroxysmal AF compared to control samples (Figure 5B). The gray module was upregulated in paroxysmal AF compared to control samples, but downregulated in persistent AF samples compared to paroxysmal AF samples (Figure 5C). Of note, our results indicated that turquoise module was significantly positively correlated to the progression of AF, upregulated in paroxysmal and persistent AF compared to control samples, and upregulated in persistent AF samples compared to paroxysmal AF samples (Figure 5D). Finally, our results showed that yellow module was upregulated in persistent AF and paroxysmal AF samples compared to control samples, and not differently expressed in persistent AF compared to paroxysmal AF samples (Figure 5E). These results suggest that blue module and turquoise module may have a crucial role in the occurrence and progression of AF, that the brown module may have a more important role in the progression of AF, and that the yellow module may have a more important role in the occurrence of AF.

### Identification of Hub Genes

**FIGURE 6:**
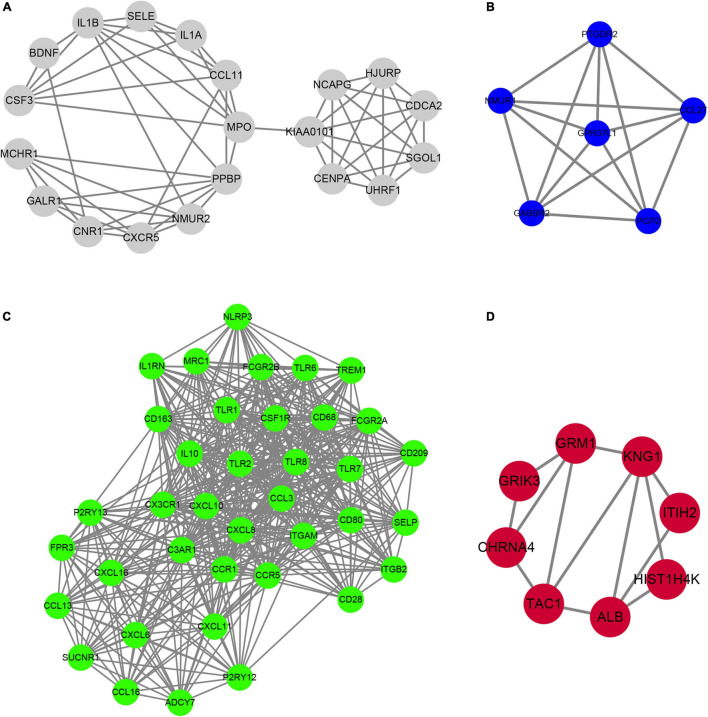
Construction of hub PPI networks in the hub modules. **(A–D)** The hub PPI network was constructed in gray module **(A)**, blue module **(B)**, turquoise module **(C)**, and brown module **(D)**.

In order to determine the hub-mRNAs associated with AF, we built a PPI network and used the MCODE scoring system. A total of 4 hub networks were identified in the AF related modules. As presented in Figure 6, a hub network including 19 nodes and 57 edges were identified in gray module (Figure 6A); a hub network including 6 nodes and 15 edges were identified in blue module (Figure 6B); a hub network including 38 nodes and 464 edges were identified in turquoise module (Figure 6C); a hub network including eight nodes and 13 edges were identified in grown module (Figure 6D).

### The Construction of lncRNA–mRNA Regulatory Network

**FIGURE 7:**
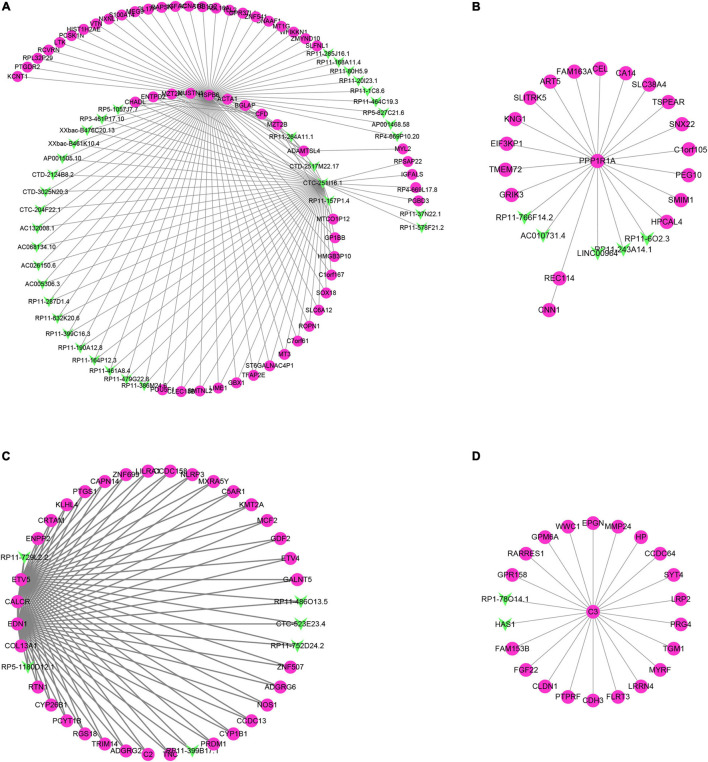
Construction of lncRNA-mRNA interaction network in the hub modules. **(A–D)** The lncRNA-mRNA interaction network was built in blue module **(A)**, brown module **(B)**, turquoise module **(C)**, and yellow module **(D)**.

Through the correlation analysis of the DEG and differentially expressed lncRNA (DEL) of the turquoise module, the brown module and all the green and yellow modules, we constructed the DEG-DEL co-expression network. As shown in Figure 7, the blue module includes 35 lncRNAs and 51 mRNAs; the brown module includes five lncRNAs and 19 mRNAs; the turquoise module includes six lncRNAs and 34 mRNAs; and the yellow module includes two lncRNAs and 21 mRNAs.

Therefore, we found that some DEGs and DElncRNAs act as hub regulators (connection degree ≥ 5) in the lncRNA-mRNA regulatory network. For example, HSPB6 in the blue module interacts with 11 LncRNAs while CTC-251I16.1 in the blue module connects more than 10 mRNAs and 20 lncRNAs; in the brown module PPP1R1a connects 17 mRNAs and 5 lncRNAs; EDN1, CALCR, COL13A1, and ETV5 in the turquoise module act as the hub genes and connect with more than 30 mRNAs and five lncRNAs; C3 in the yellow module is connected to 20 mRNAs and 2 lncRNAs.

### The Function Prediction of Hub Genes and IncRNAs of Turquoise Module

**FIGURE 8:**
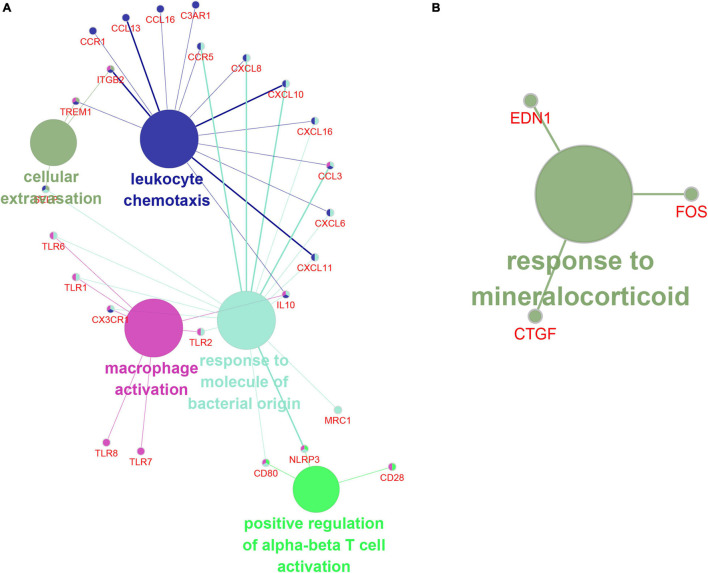
The function prediction of hub gene and lncRNA of turquoise module. **(A,B)** The function prediction of Hub mRNAs **(A)** and lncRNAs **(B)** of turquoise module.

In order to understand the biological effects of hub genes and hub lncRNAs, we performed function prediction via the plug-in CLUEGO in Cytoscape. Our results showed that hub genes in turquoise module was significantly related to cell extravasation, leukocyte chemotaxis, macrophage activation, response to bacteria-derived molecules and positive regulation of α-β T cell activation (Figure 8A). Next, we predicted the potential function of hub lncRNA to in the turquoise module using its co-expressing mRNA. The hub lncRNA in the turquoise module was significantly related to the mineralocorticoid response (Figure 8B).

## Discussion

Atrial fibrillation/paroxysmal AF (PAF) is the main cause of cardiogenic embolism. In recent years, the progression from paroxysmal AF to persistent AF has attracted more and more attention (Proietti et al., 2015). Emerging data indicated a significant association between the morbidity and the transition. However, the molecular mechanism of the progression of AF is unclear. Therefore, we performed RNA sequencing for normal samples, paroxysmal AF and persistent AF samples to identify DEG and explore the roles of these DEGs in AF. In our current research, it is very interesting that we found that the expression of various lncRNAs in the progression of atrial fibrillation was differently changed. Totally, 272 DEmRNAs and 286 DElncRNAs were identified in paroxysmal atrial fibrillation compared to normal samples; 324 DEmRNAs and 258 DElncRNAs found in persistent atrial fibrillation compared with normal samples; and 520 DEmRNAs and 414 DElncRNAs were identified to be differently expressed in persistent AF compared to paroxysmal AF samples (Figures 1E,F). Among the DEGs, about 50% were coding genes and around 50% were non-coding RNAs, suggesting that lncRNAs may also have a crucial role in the progression of AF.

Considering that potential functions of these DEmRNAs in AF remained to be unclear, we performed the enrichment analysis of DEGS. we found that DEGs were involved in the regulation of multiple biological processes and pathways in the progression of AF. It is worth noting that the DEGs between normal and paroxysmal atrial fibrillation was most related to the regulation of VEGF production and the binding to the CXCR chemokine receptor. The VEGF-VEGFR system is essential in angiogenesis and lymphangiogenesis (Shibuya, 2015). Studies have shown that VEGFs play a crucial role in the occurrence, and development of AF (Chung et al., 2002). Multiple studies in patients with early atrial fibrillation reported elevated levels of VEGFA. Vascular endothelial growth factor promotes atrial arrhythmia by inducing acute intercalary disk remodeling (Mezache et al., 2020). It is very interesting that our research is consistent with previous reports that intracardiac VEGF levels increased in patients with paroxysmal, but not persistent AF (Scridon et al., 2012). CXCR family members also play an important role in AF. For example, the chemokine receptor CXCR-2 is a key regulator of monocyte mobilization in hypertension and heart remodeling; and blocking the activation of CXCR-2 can be used as a new treatment strategy for AF (Zhang et al., 2020). We also revealed that DEG between normal and sustained AF was most significantly correlated with mucin-type O-glycan biosynthesis. So far, our research reveals the relationship between this pathway and AF for the first time. Finally, we found that DEG between paroxysmal and persistent atrial fibrillation was most significantly associated with the interleukin-18, coagulation, and complement cascade. Interleukin-18 plays a central role in the regulation of both innate and adaptive immunity (Dinarello, 2018). A previous study showed that AF patients have higher levels of IL-18. IL-18 is positively related to the inner diameter of the left atrium (Luan et al., 2010). A recent genetic study showed that genetic variation of interleukin-18 is related to a lower risk of atrial fibrillation among people in the Northeast China (Wang et al., 2017). In addition, Kornej et al. (2018) reported that complement and coagulation cascades were also related to AF. These reports, together with our findings, further prove the key role of these signals in AF.

Weighted gene co-expression network analysis has been applied to identify the core genes in AF. For example, Zou et al. (2018) reported that LEP, FOS, EDN1, NMU, CALB2, TAC1 may be related to the occurrence and maintenance of AF using WGCNA method and public dataset GSE41177. Li et al. (2020) used GSE79768 to perform a WGCNA analysis to determine the key modules related to atrial fibrillation. However, these reports were based on online public databases and the clinical information of the samples used in these reports remained unclear. In this study, we collected 10 control samples, 10 paroxysmal AF and 10 persistent AF to perform RNA-sequencing analysis. Moreover, we performed PPI network and WGCNA analysis to reveal the biological mechanisms related to the progression of atrial fibrillation. Five modules were identified in the progress of AF, including blue, brown, gray, turquoise, and yellow modules. By analyzing the correlation between these modules and the progression of atrial fibrillation, we found that the turquoise module was significantly positively correlated with the progression of atrial fibrillation while the blue module was significantly negatively correlated with the progression of AF. Moreover, a PPI network was used to identify functional gene connections through MCODE based on a scoring system. Several hub genes were also found in different modules, such as KIAA0101, UHRF1, CDCA2, HJURP, NCAPG, SGOL1, and CENPA in grey module, GPR37L1 in blue module, CD163, CD28 and CX3CR1 in turquoise module, and KNG1 and GRM1 in brown module. It is worth noting that several of these hub genes are reported to be significantly associated with AF. For example, interleukin 10 treatment improves inflammatory atrial remodeling and fibrillation induced by a high-fat diet (Kondo et al., 2018). The plasma concentration of IL-10 in the acute phase is associated with high risk sources of cardiogenic stroke. The serum soluble CD163 level in AF was significantly higher than that in patients with sinus rhythm (Zhong et al., 2016).

Recently, accumulating evidence indicates a link between immune response and AF (Liu et al., 2018). Previous studies demonstrated that marcrophages lead to both structure and electric atrial remodeling in AF (Liu et al., 2018). In addition, a large number of studies have shown that T cells are closely related to cardiovascular diseases, including AF (Liu et al., 2018). For instance, a significant high CD4 + CD28null T cells was found in patient with AF (Sulzgruber et al., 2017). However, the precise mechanism remains unclear. In this study, we conducted a bioinformatics analysis on the turquoise module and reported that the Hub PPI network in the turquoise module significantly participates in regulating leukocyte chemotaxis and macrophage activity and positively regulates α-β T cell activation, indicating that immune pathways have a key role in AF.

LncRNA plays an important role in the regulation of cardiovascular diseases. LncRNA HOTAIR, as a ceRNA, regulates the remodeling of connexin 43 during AF by sponging microRNA-613 (Dai et al., 2020). LncRNA TCONS-00106987 stimulates miR-26 to regulate KCNJ2 to promote atrial electrical remodeling during AF (Du et al., 2020). LncRNA-LINC00472 reduces the expression of JP2 and RyR2 through miR-24, thereby participating in the pathogenesis of AF (Wang et al., 2019). In this study, we identified 286 DElncRNAs in paroxysmal AF and 258 DElncRNAs in persistent AF. Also 414 DElncRNA were identified between persistent and paroxysmal AF samples. Of note, several lncRNAs were observed to differently express in multiple stages of AF, such as MTND1P23, RP11-1081M5.2, XIST and BANCR. Interestingly, several of them had been reported to have key roles in human disease, such as XIST and BANCR. For example, BANCR was previously identified as a cancer-promoting lncRNA and was also significantly related to the pathogenesis of multiple cardiovascular diseases (Li et al., 2017; Wilson et al., 2020). For example, Wilson et al. (2020) found that BANCR promotes cardiomyocyte migration in humans. BANCR promotes vascular smooth muscle cell proliferation via JNK pathway (Li et al., 2017). XIST has a regulatory role in cardiomyocyte function, modulates cardiomyocyte apoptosis via miR-873 (Cai et al., 2020), promotes cardiac fibroblasts proliferation by sponging miR-155-5p (Zhang et al., 2021), and protects the hypoxia-induced cardiomyocyte injury via HK2 (Fan et al., 2020), indicating that XIST’s key roles in heart development. Also, in this study we identified several central lncRNA-mRNA co-expression networks during AF, such as CTC-251I16.1 in the blue module, which connects to more than 10 mRNAs and more than 20 lncRNAs. Bioinformatics analysis showed that the central lncRNA in the turquoise module is significantly related to the mineralocorticoid response.

Our research has several limitations. First, the size of clinical samples used in this study was limited. In the future study, we plan to collect more AF samples and comprehensive clinical information to confirm the correlation between hub genes and AF progression. Secondly, we did not perform gain/loss of function analysis to explore the potential role of hub gene and lncRNA in AF.

In this study, the WGCNA algorithm was for the first time to systematically explore the roles of DEGs in AF. Bioinformatics analysis demonstrated that these DEGs were significantly related to regulate multiple AF related pathways, such as the regulation of VEGF production and binding to the CXCR chemokine receptor. Furthermore, five hub modules were identified in the progression of AF, including blue, brown, gray, turquoise and yellow modules. These results provide new information for further understanding of the pathogenesis and differential diagnosis of AF.

## Data Availability Statement

The data presented in the study could be accessed by requesting to the corresponding author.

## Ethics Statement

The studies involving human participants were reviewed and approved by the Chongqing General Hospital, University of Chinese Academy of Sciences. The patients/participants provided their written informed consent to participate in this study.

## Author Contributions

HC designed the project. PY and YC performed bioinformatics analysis. PY and HJ prepared the manuscript. All authors read and approved the final manuscript.

## Conflict of Interest

The authors declare that the research was conducted in the absence of any commercial or financial relationships that could be construed as a potential conflict of interest.

## Publisher’s Note

All claims expressed in this article are solely those of the authors and do not necessarily represent those of their affiliated organizations, or those of the publisher, the editors and the reviewers. Any product that may be evaluated in this article, or claim that may be made by its manufacturer, is not guaranteed or endorsed by the publisher.
